# Supplementary material for: Tobacco rattle virus-induced PHYTOENE DESATURASE (PDS) and Mg-chelatase H subunit (ChlH) gene silencing in Solanum pseudocapsicum L
Source: PeerJ. 2018 Mar 20;6:e4424. doi: 10.7717/peerj.4424 (PMC5865466; doi:10.7717/peerj.4424)
Supplement: Supplemental Information 1 — Fig. S1. Alignment of the sequencing results of TRV2-SpPDS and the inserted PDS fragments. C–A, sequence of TRV2-SpChl H. C-B, sequence of insert ChlH fragment. Fig. S2. Alignment of the sequencing results of TRV2-SpChlH and the inserted ChlH fragments. P-A, sequence of TRV2-SpPDS. P-B, sequence of insert PDS fragment. Table S1. Primers used in this study. [file peerj-06-4424-s001.docx]

**Supplementary information**

**Fig. S1. Alignment of the sequencing results of TRV2-Sp*PDS* and the inserted *PDS* fragments**

P-A, sequencing result of TRV2-*SpPDS.* P-B, sequencing result of insert *PDS* fragment. Alignment of the sequencing results of TRV2- *SpPDS* (P-A) and the inserted *PDS* (P-B) fragment showed that the two sequences shared 100% similarity.

**Fig. S2. Alignment of the sequencing results of TRV2-Sp*ChlH* and the inserted *ChlH* fragments**

C-A, sequencing result of TRV2-*SpChlH.* C-B, sequencing result of insert *ChlH* fragment. Alignment of the sequencing results of TRV2- *SpChlH* (C-A) and the inserted *ChlH* (C-B) fragment showed that the two sequences shared 100% similarity.

**Supplementary Table S 1. Primers used in this study.**

| **Primers name** | **Primer sequence (5´-3´)** |
| --- | --- |
| SpACTIN-F  Sp ACTIN-R | 5´-CAGCCACTCGTCTGTGATAAT-3´  5´-TAACCACGCTCCGTCAAGAT-3´ |
| SpGAPDH-F  SpGAPDH-R | 5´-TCTCAAACCTACCGCCTCCC-3´  5´-TCGTTCACTCCCACCACAAA-3´ |
| sSpUBQ-F  SpUBQ-R  SpPDS-1-F | 5´-TTGGCAAGCAACAATCAT-3´  5´-GCAGATGGACAGCAGGAC-3´  5´-GTGGGTATCTCGCAAAGGGC-3´ |
| SpPDS-1-R  SpChlH-F  SpChlH-R  SpPDS-GSP-F | 5´-AGTCACTGCGAGATACCCAC-3´  5´-CAGCAGTCCAAATCTTAACGTA -3´  5´-ATGGTATTCGCATAACTCCTTC-3´  5´-CTGGAGAAATTAGTCGGAGTACCTG-3´ |
| SpPDS-nest-F  Oligo dT Primer  Nested Primer  TRV2_insert_yz-F  TRV2_insert_yz-R  PDS_insert_EcoRI-F  PDS_insert_EcoRI-R  ChlH_insert_SmaI-F  ChlH_insert_SmaI-R  pTRV1-F  pTRV1-R  pTRV2-F  pTRV2-R | 5´-AATTGAGGGAGATGCGTTTGTG-3´  5´-GCTGTCAACGATACGCTACGTAACGGCATGACAGTG(T)_24_-3´  5´-CGCTAGTAACGGCATGACAGTG-3´  5´-TAGATAATGGTTTGGTGGTC-3´  5´-TAGTTTAATGTCTTCGGGAC-3´  5´-TTCTGTGAGTAAGGTTACCGGCCATGTCAAAGGCACTTAA-3´  5´-CCCATGGAGGCCTTCTAGAGAGTCACTGCGAGATACCCAC-3´  5´-CACGCGTCTC GAGGCCCCAGCAGTCCAAATCTTAACGTA-3´  5´-TAATGTCTTC GGGACATGCC CATGGTATTCGCATAACTCCTTC-3´  5´-AGCAGCAACCGACGACTT-3´  5´-ACCAACTCCTTCTTCTCAGACT-3´  5´-ATATTCCTGCGAATCCAAACAC-3´  5´-GAA ACTCAAATGCTACCAACGA-3´ |
